# Function and X-Ray crystal structure of *Escherichia coli* YfdE

**DOI:** 10.1371/journal.pone.0067901

**Published:** 2013-07-23

**Authors:** Elwood A. Mullins, Kelly L. Sullivan, T. Joseph Kappock

**Affiliations:** 1 Department of Biochemistry, Purdue University, West Lafayette, Indiana, United States of America; 2 Department of Chemistry, Washington University in St. Louis, St. Louis, Missouri, United States of America; University of Florida, United States of America

## Abstract

Many food plants accumulate oxalate, which humans absorb but do not metabolize, leading to the formation of urinary stones. The commensal bacterium *Oxalobacter formigenes* consumes oxalate by converting it to oxalyl-CoA, which is decarboxylated by oxalyl-CoA decarboxylase (OXC). OXC and the class III CoA-transferase formyl-CoA:oxalate CoA-transferase (FCOCT) are widespread among bacteria, including many that have no apparent ability to degrade or to resist external oxalate. The EvgA acid response regulator activates transcription of the *Escherichia coli yfdXWUVE* operon encoding YfdW (FCOCT), YfdU (OXC), and YfdE, a class III CoA-transferase that is 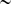
30% identical to YfdW. YfdW and YfdU are necessary and sufficient for oxalate-induced protection against a subsequent acid challenge; neither of the other genes has a known function. We report the purification, in vitro characterization, 2.1-Å crystal structure, and functional assignment of YfdE. YfdE and UctC, an orthologue from the obligate aerobe *Acetobacter aceti*, perform the reversible conversion of acetyl-CoA and oxalate to oxalyl-CoA and acetate. The annotation of YfdE as acetyl-CoA:oxalate CoA-transferase (ACOCT) expands the scope of metabolic pathways linked to oxalate catabolism and the oxalate-induced acid tolerance response. FCOCT and ACOCT active sites contain distinctive, conserved active site loops (the glycine-rich loop and the GNxH loop, respectively) that appear to encode substrate specificity.

## Introduction

**Figure 1:**
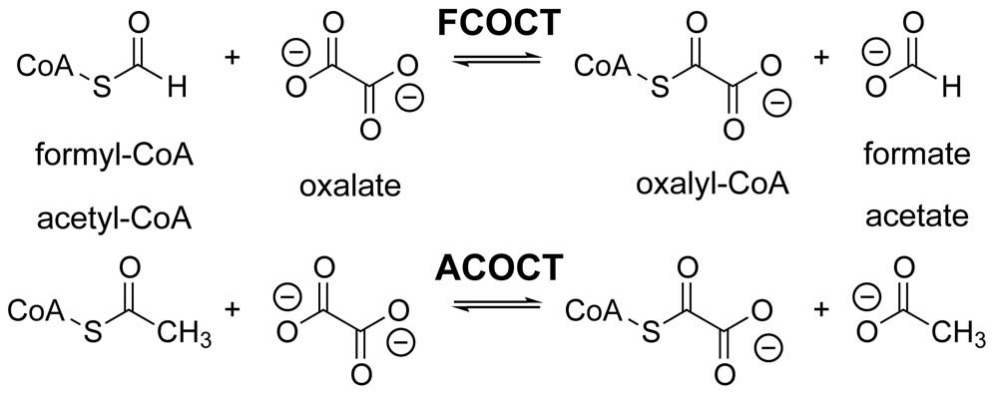
Class III CoA-transferase reactions. FCOCT and ACOCT reactions.

Humans cannot catabolize oxalate, which promotes calcium oxalate urinary stone formation and other maladies [1]. Edible plants, some of which contain 80% oxalate (dry weight) [2], are the main sources of oxalate. Dietary oxalate uptake is suppressed by commensal oxalate degraders like *Oxalobacter formigenes* [3], [4], an anaerobic gut bacterium that derives energy and carbon from a concise oxalate catabolic pathway centered on oxalyl-CoA decarboxylase (OXC) [5]. Thiamin-dependent OXC converts oxalyl-CoA and a proton to formyl-CoA and 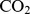 [6], [7] in a singular 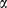-decarboxylation reaction that is specific for oxalyl-CoA [6], [8]. The class III CoA-transferase [9] formyl-CoA:oxalate CoA-transferase (FCOCT, encoded by *frc*), regenerates oxalyl-CoA with concomitant production of formate (Figure 1) [10], [11]. Together, FCOCT and OXC raise cytoplasmic pH [12], and in combination with the electrogenic oxalate:formate antiporter OxlT, generate a proton gradient [5].

**Figure 2:**
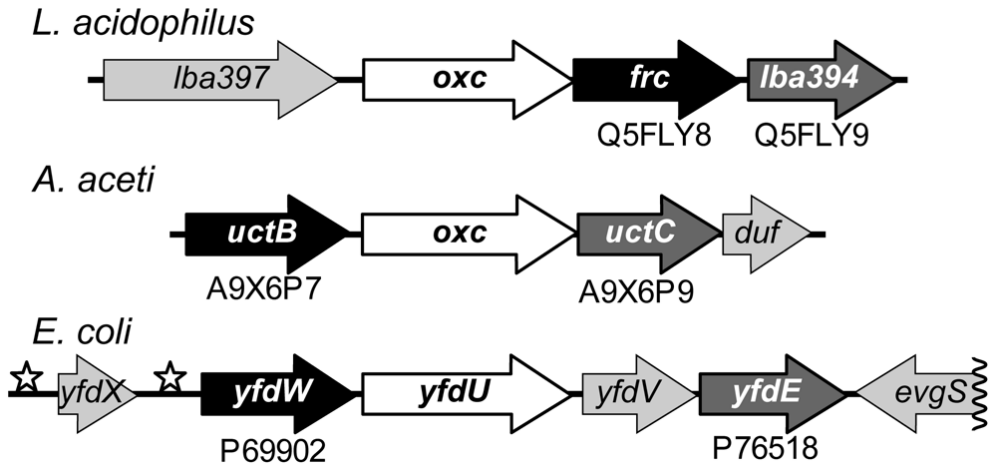
Class III CoA-transferases associated with oxalate metabolism. Selected gene clusters that contain *oxc* (white backgrounds) and two class III CoA-transferase genes. CoA-transferase gene names are shown in white: black backgrounds, FCOCT subgroup; medium-gray backgrounds, ACOCT subgroup. UniProt accession numbers are given for each CoA-transferase. Unrelated proteins have light gray backgrounds; flanking proteins on the opposite DNA strands are not shown, except for the 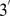 end of the *E. coli evgS* gene, the sensor kinase of the EvgAS two-component response regulator [14]. Stars indicate the locations of inverted repeats to which EvgA binds, inducing the expression of *yfdX* and *yfdWUVE* [15].

*Escherichia coli* and many other bacteria that do not consume or even tolerate oxalate nevertheless possess FCOCT and OXC [13]. Transcription of the *E. coli yfdXWUVE* operon (Figure 2) containing *yfdW* (FCOCT) and *yfdU* (OXC) is activated by the acid-response regulator EvgA [14], [15]. EvgA activates proton-consuming amino acid decarboxylases during strong acid resistance responses [16]. Oxalate catabolism might similarly counteract acid stress by oxalyl-CoA decarboxylation. However, the source of the cytoplasmic oxalate that is presumably required is not always apparent.

Bacteria exposed to oxalate exhibit improved survival during subsequent acid challenges, a process associated with the induction of *yfdW* homologues [17], [18]. Oxalate elicits a moderate, *rpoS*-independent acid tolerance response (ATR) that requires both *yfdW* and *yfdU* in *E. coli* [19]. YfdV and YfdE appear to have a secondary role in the oxalate-induced ATR. Insight into molecular basis for the ATR might be gained by establishing functions for YfdX, a putative membrane protein, YfdV, an unassigned membrane transport protein, and YfdE, a class III CoA-transferase [9] that resembles YfdW.

The sequence similarity and syntenic relationship of *yfdW* and *yfdE* suggested that they might share an acyl-CoA substrate. This hypothesis was tested with functional gene expression studies of YfdE and UctC, a YfdE homologue from *Acetobacter aceti*. We demonstrate that these proteins have acetyl-CoA:oxalate CoA-transferase (ACOCT) activity, which allows a core metabolite to serve as an alternate source of oxalyl-CoA. A crystal structure of YfdE fused to an N-terminal hexahistidine tag (H6YfdE) reveals that a conserved GNxH motif replaces the glycine-rich loop previously associated with FCOCT substrate selection [20].

## Results

### Paired bacterial CoA-transferase genes located near *oxc*

Bacterial *oxc* genes are often, but not always [7], [11], located near a class III CoA-transferase gene resembling *yfdW*. Some bacterial *oxc* genes have syntenic relationships to two *yfdW* homologues, each of which appears to encode an FCOCT isozyme [21], [22]. In *E. coli* [14] and other enterobacteria, the lactic acid bacterium *Lactobacillus acidophilus* [17], and the acetic acid bacterium *A. aceti* 1023 [23], *oxc* is located near two *different* class III CoA-transferase genes: one resembling *yfdW* and the other resembling *yfdE* (Figure 2). *yfdU* (*oxc*), *yfdW*, and *yfdE* are co-expressed as part of the *E. coli yfdXWUVE* operon [14].

### Protein expression, purification, and characterization

YfdE, UctC, and variants of each protein were overexpressed in *E. coli* BL21(DE3) or C41(DE3) and purified to 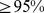 homogeneity, with the exception of YfdE (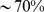 pure). Electrospray ionization-mass spectrometry analysis was consistent with the expected protein masses (Table S1). The mass spectrum for YfdE fused to a C-terminal hexahistidine tag (YfdEH6) also contained a minor peak corresponding to the aspartyl-CoA thioester adduct formed during class III CoA-transferase reactions [24]. Analytical size exclusion chromatography indicated that H6YfdE and UctC are dimers in solution (Figure S1).

### Substrate identification and kinetic characterization

**Figure 3:**
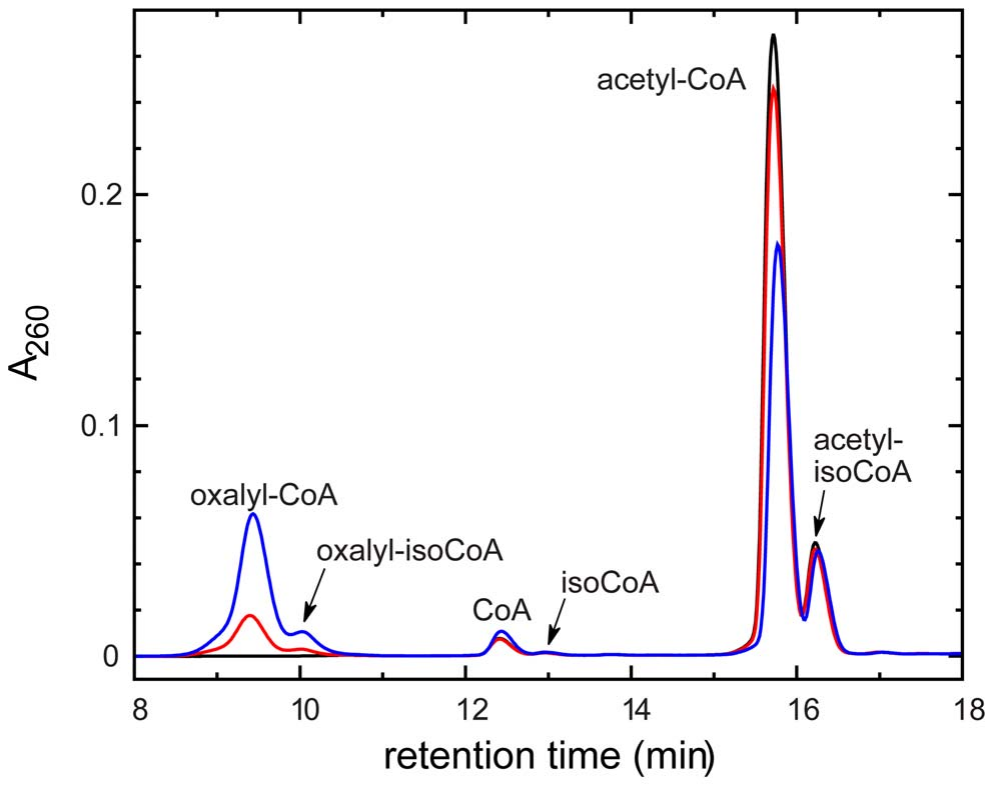
HPLC analysis of ACOCT reaction progress. HPLC traces showing time-dependent conversion of acetyl-CoA to oxalyl-CoA by UctC. IsoCoA is the 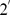-phosphoryl isomer of CoA [49]. Black trace, 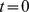; red trace, 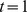 min; blue trace, 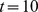 min.

A previously described high performance liquid chromatography (HPLC) method [23], [25] was used to identify substrates for YfdE and UctC. Candidate substrate pairs were incubated with enzyme, then acid-quenched and analyzed by HPLC (Figure 3). All HPLC analyses were performed promptly relative to the hydrolysis rates of the acyl-CoAs under study (Figure S2). Both YfdE and UctC produced oxalyl-CoA from acetyl-CoA/oxalate (Table 1). Both also produced acetyl-CoA and oxalyl-CoA from formyl-CoA/acetate and formyl-CoA/oxalate, respectively, albeit with significantly reduced specific activities. Neither produced a detectable acyl-CoA product from acetyl-CoA/succinate, formyl-CoA/succinate, or succinyl-CoA/oxalate.

**Table 1:** Identification of substrates for YfdE and UctC.

		Specific activity^a^ (units mg 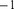 )	
Acyl-CoA	Carboxylate	YfdE^b^	UctC
acetyl-CoA	oxalate	9.5	1.4
	succinate	NR^c^	NR^c^
formyl-CoA	acetate	1.9	0.12
	oxalate	1.5	0.10
	succinate	NR^c^	NR^c^
succinyl-CoA	oxalate	NR^c^	NR^c^

a A unit of enzyme synthesizes one 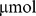 of acyl-CoA product per min. Product peaks were identified and quantitated by HPLC.

b No correction was applied to account for the lower purity of YfdE (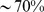).

c NR, no reaction product detected. The detection threshold was approximately 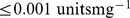.

Kinetic parameters were determined for YfdE, H6YfdE, YfdEH6, and UctC by monitoring the rate of oxalyl-CoA formation in the presence of a fixed concentration of either acetyl-CoA or oxalate (Table 2 and Figure S3). All variants of YfdE exhibited comparable kinetic properties, while UctC had higher apparent oxalate affinity but a lower turnover number (Table 2). UctC-D177A failed to support detectable activity (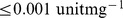), confirming that Asp177 (YfdE Asp173) is the site of covalent catalysis.

**Table 2:** Kinetic parameters for YfdE and UctC.^a^

Substrate	Parameter	YfdE^b^	H6YfdE	YfdEH6	UctC
acetyl-CoA^c^	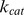 ( 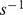 )	11	12	6.9	2.0
	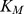 ( 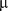 M)	17	18	13	81
	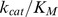 ( 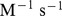 )	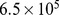	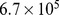	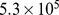	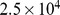
oxalate^d^	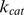 ( 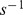 )	15	22	10	1.8
	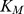 (mM)	22	39	22	1.0
	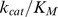 ( 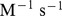 )	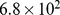	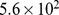	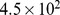	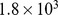

a Saturation curves are shown in the Supporting Information (Figure S3). 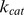 values were computed assuming one active site per subunit and individual subunit masses (Table S1).

b No correction was applied to account for the lower purity of YfdE (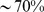).

c Determined at 50 mM oxalate. This concentration is subsaturating for YfdE forms; the 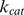 and 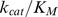 values given are likely to be lower than the true values.

d Determined at 0.75 mM acetyl-CoA.

YfdE and UctC appear to function principally as ACOCTs (Figure 1), with turnover numbers and substrate promiscuity similar to that of other class III CoA-transferases [12].

### X-ray crystal structure of H6YfdE

The structure of H6YfdE was solved by molecular replacement using a biochemically uncharacterized protein from *Brucella suis* (Tables 3 and 4). Six subunits, forming three dimers, were placed in the asymmetric unit. Electron density for dimers AB (Figure S4) and CD was superior to that for dimer EF, but no significant differences in the resulting models were noted. In all subunits, the N-terminal appendage and the first five to six residues were disordered. The last residue (Ser381) was also disordered in some subunits. No ligands or covalent adducts were present.

**Table 3:** Crystallographic data collection statistics for H6YfdE.^a^

Beamline	21-ID-G
Wavelength (Å)	0.97856
Space group	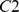
Unit cell dimensions	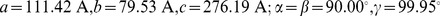
Resolution (Å)	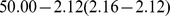
No. measured reflections	341,096
No. unique reflections	107,843 (4,956)
Completeness (%)	79.8 (73.7)^b^
Redundancy	3.2 (3.1)
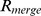 c (%)	8.0 (50.6)
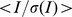	16.2 (2.2)
Wilson *B*-factor ( 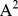 )	25.97
Matthews coefficient ( 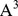 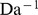 )	2.29
Solvent content (%)	46

a Values in parentheses are for the highest resolution shell.

b Lower completeness at <4 Å appears to have been caused by a blind spot, but anisotropic diffraction has not been unambiguously excluded.

c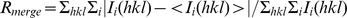, where 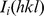 is the intensity of the 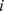
th observation of reflection 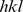 and 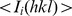 is the mean intensity of all observations 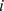 of reflection 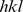.

**Table 4:** Crystallographic refinement statistics for H6YfdE.^a^

Resolution (Å)	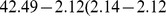 )
No. reflections in working set	102,367 (3,064)
No. reflections in test set	5,455 (170)
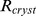 b (%)	18.9 (23.2)
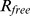 c (%)	23.6 (30.3)
No. protein atoms	17,260
No. water atoms	1,021
Average B-factor ( 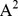 )	
Protein atoms	32.8
Water atoms	33.8
Rmsd	
Bonds (Å)	0.003
Angles (  )	0.8
Ramachandran plot (%)	96.78, 3.22, 0.00
Rotamer outliers (%)	1.88
Clash score^d^	8.05
PDB id	4hl6

a Values in parentheses are for the highest resolution shell.

b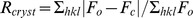, where 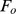 and 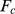 are the observed and calculated structure factors, respectively.

c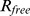 was calculated in the same manner as 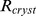 using the 5% of reflections excluded from refinement.

d Number of serious steric overlaps (>0.4 Å) per 1000 atoms [74].

**Figure 4:**
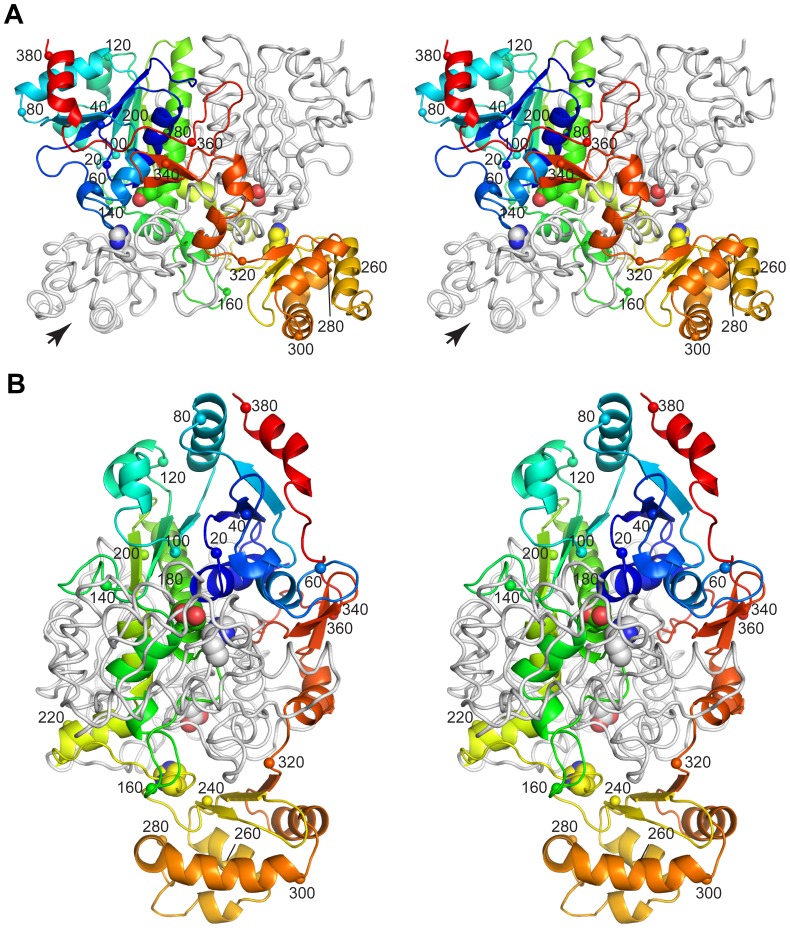
Stereogram of the H6YfdE dimer. One subunit is shown as a rainbow-colored cartoon where the color gradient corresponds to the sequence; the small domain is at the bottom. The other subunit is shown as a light gray ribbon. Small spheres and numbers identify the 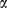-carbon at intervals of 20 residues. The active site residues Asp173 and His233 (large spheres) are provided by different subunits in each active site. A topology diagram is provided in the Supporting Information (Figure S5). (A) View with the pseudo-twofold axis relating the subunits vertical in the plane of the page. Spheres corresponding to residues 220 and 240 are hidden by the partner subunit. (B) View along the long axis of one subunit as indicated by the large arrow in panel A.

**Figure 5:**
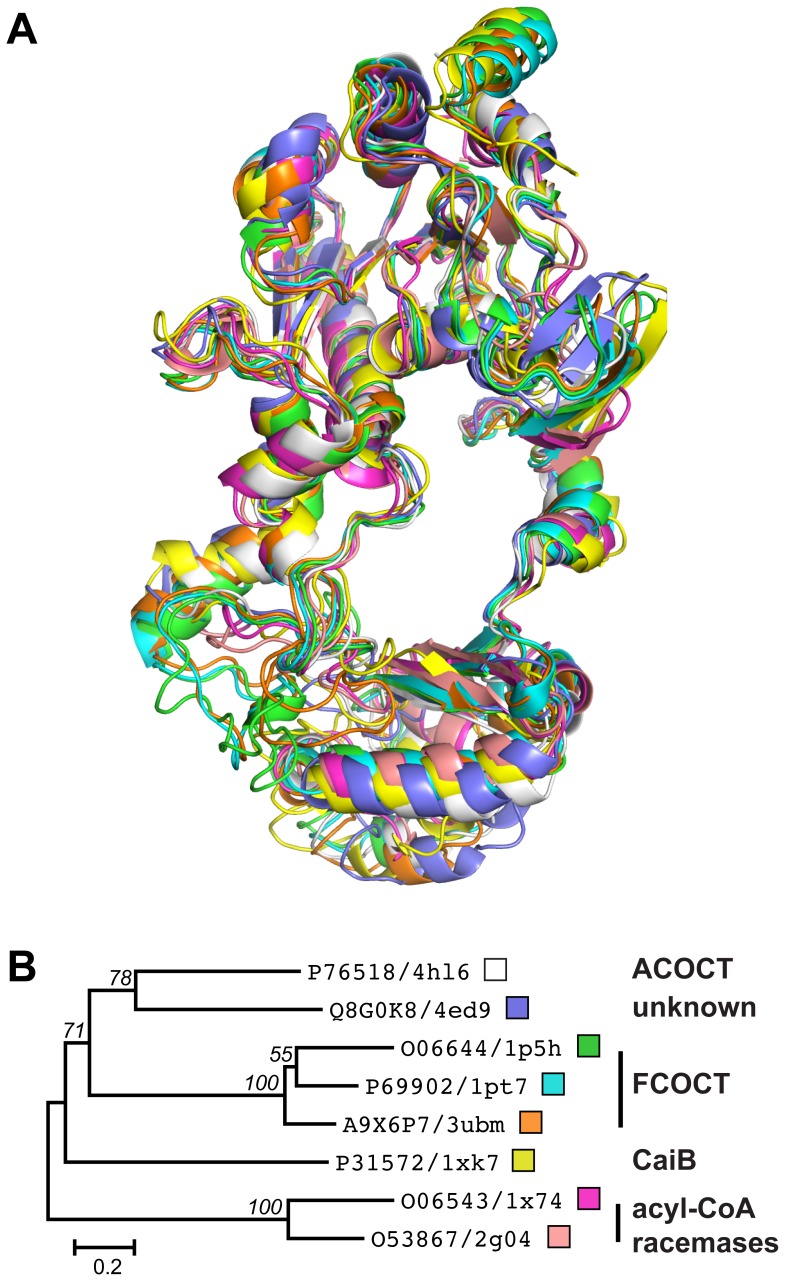
Structural comparison of class III CoA-transferase superfamily members. (A) Structure superposition of eight class III CoA-transferase superfamily members, shown in the same orientation as Figure 4B. PDB entries are 4hl6 (white), 4ed9 (dark blue), 1p5h (green) [69], 1pt7 (cyan) [24], 3ubm (orange) [25], 1×k7 (yellow) [70], 1×74 (magenta) [71], and 2g04 (pink) [72]. (B) ML phylogram of the structure-based sequence alignment (Figure S6) that corresponds to panel A. UniProt accession numbers and PDB entry codes are given for each taxon. The small boxes match ribbon colors in panel A. Nodes in this consensus tree with 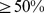 support in 1000 bootstrap replicates are labeled. The scale bar is in units of amino acid substitutions per site.

The monomer topology of H6YfdE (Figure 4 and Figure S5) closely resembles that of other class III CoA-transferase superfamily members (Figure 5). Each subunit is composed of a small domain and a large domain. The large domain is formed by residues in both N-terminal and C-terminal regions of the polypeptide chain, creating a central hole through which the other subunit is threaded. This interlocked arrangement creates an extensive monomer-monomer interface (4160 Å^2^) that accounts for 30% of the surface area of each H6YfdE subunit. [The small dimer-dimer interface (∼120 Å^2^) is not likely to be physiologically relevant.] Structural differences between H6YfdE and other class III CoA-transferase superfamily members are primarily due to loop length variations and rotation of the small domain. In structure-based sequence alignments, YfdE is 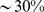 identical to YfdW and other FCOCTs (Table S2).

**Figure 6:**
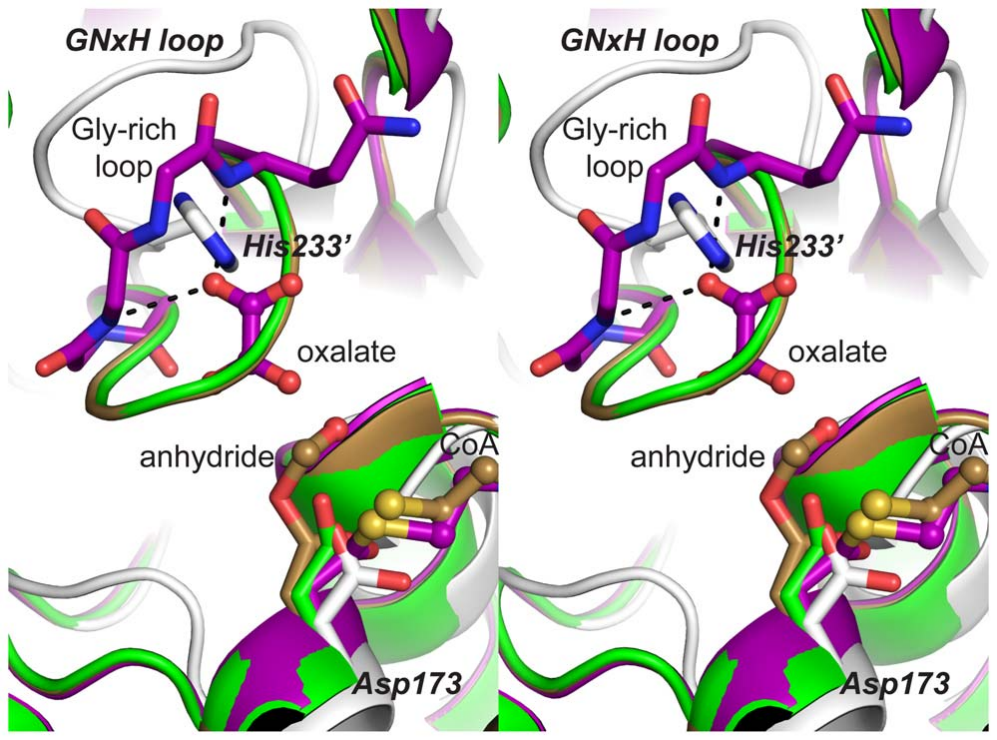
Stereogram of FCOCT and ACOCT active sites. Superposition of H6YfdE structure (gray, PDB entry 4hl6) on several *O. formigenes* FRC structures: apo (green, PDB entry 1p5h) [69], aspartylformyl anhydride adduct plus CoA (light brown, PDB entry 2vjm) [20], and aspartyl-CoA thioester adduct plus oxalate (purple, PDB entry 2vjo) [20]. Bold italic letters identify significant features in the H6YfdE active site.

**Figure 7:**
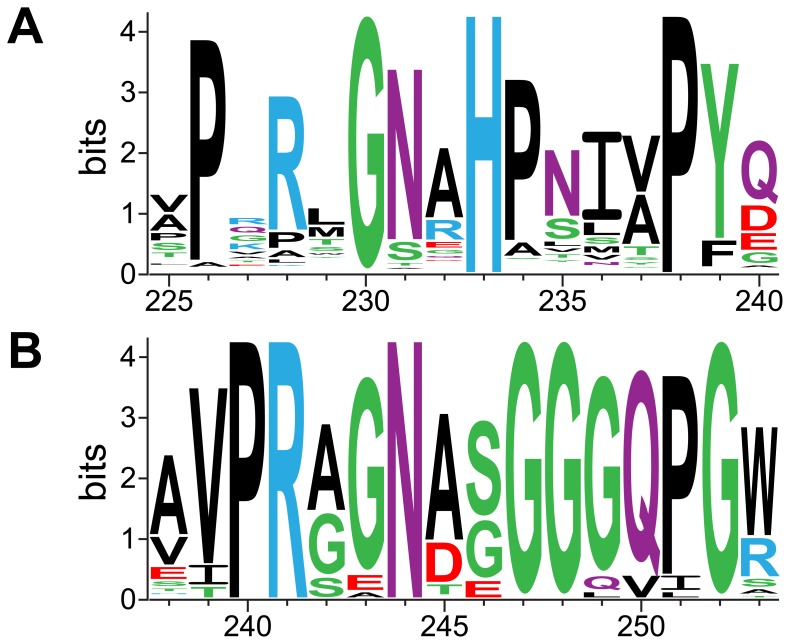
Sequence logos for FCOCT and ACOCT active site loops. (A) Sequence logo for the GNxH loop region in 1116 bacterial homologues of *yfdE*. The height of each residue code corresponds to the degree of conservation at the indicated position. YfdE sequence numbers are given at the bottom. (B) Sequence logo for the glycine-rich loop region in 182 bacterial homologues of *yfdW*. The height of each residue code corresponds to the degree of conservation at the indicated position. YfdW sequence numbers are given at the bottom.

The YfdE active site, positioned at the interface of the small domain of one subunit and the large domain of the partner subunit, was located by structure superpositions with liganded and adducted FCOCT crystal structures (Figure 6). The only conserved polar residue in the ACOCT family that appears capable of interacting with bound carboxylate substrates or acylaspartyl anhydride adducts is His233. 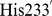 (His233 from a partner subunit) is located in a GNxH motif that forms part of a loop opposite the covalent attachment point Asp173. The GNxH loop replaces the glycine-rich loop that binds oxalate in YfdW and other FCOCTs [20]. A PHI-BLAST search identified a larger conserved motif among YfdE homologues (Figure 7) that diverges substantially from the structurally analogous motif in YfdW homologues (Figure 6 and Figure S6). These results suggest that the common substrate oxalate is not recognized in the same fashion by YfdE and YfdW. We propose that this differentially conserved sequence region can be used to discern enzyme functions within the closely related superfamily of class III CoA-transferases (Figure 5B).

## Discussion

**Figure 8:**
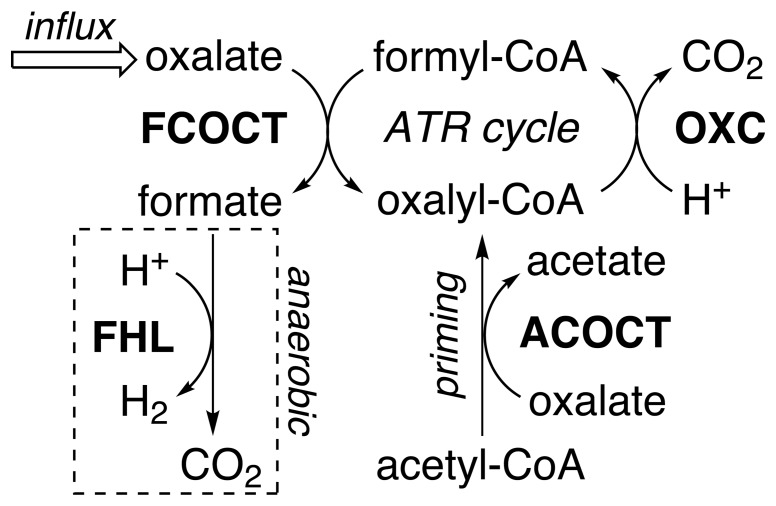
ACOCT primes the oxalate-induced ATR cycle. YfdW and YfdU appear to be required for the oxalate-induced ATR [19]. Arrows indicate the expected physiological direction; only OXC catalyzes an irreversible reaction [13], [73]. ACOCT enables the synthesis of oxalyl-CoA when formyl-CoA and formate are unavailable (e.g., aerobic conditions). The aerobic fate of formate is unclear; the FHL reaction in the dotted box is associated with anaerobic conditions. Oxalate/formate antiport is not detected in *E. coli* [19]. ACOCT and FCOCT could also work together to support the oxalate-catalyzed interconversion of formyl-CoA/acetate and acetyl-CoA/formate (not shown).

Membrane-permeant carboxylic acids suppress the growth of microbes unless a suitable resistance system is present. *E. coli* encounters constantly changing environments and diverse carboxylic acids that require multiple acid-resistance systems controlled by complex regulatory schemes [16]. The *yfdXWUVE* operon is regulated by EvgA and required for the oxalate-induced ATR [15], [19]. This study shows that YfdE is acetyl-CoA:oxalate CoA-transferase. ACOCT has been previously proposed to have a role in oxalate catabolism [26], but activity was not previously associated with a particular protein. We suggest that ACOCT is an accessory enzyme that serves to “prime” oxalate catabolism, forming oxalyl-CoA when formyl-CoA is not available (Figure 8).

### Oxalate metabolism

Oxalate is abundant in plant-derived materials, but it is also toxic, particularly at low pH. Consequently, few microbes exploit oxalate as a carbon or energy source. The specialized oxalate degrader *O. formigenes* requires a constant supply of oxalate [27] to establish a proton gradient [5], [28]. Abundant FRC supports rapid oxalyl-CoA recycling and thereby rapid oxalate catabolism. FRC is not particularly selective [12], presumably because cytoplasmic oxalate is always available.

Enterobacteria have more versatile metabolic capabilities appropriate for the dynamic environments encountered in animal digestive systems, which may or may not contain oxalate [19]. The oxalate-induced ATR requires OXC and FCOCT, but whether protection from subsequent acid exposure is due to proton uptake during oxalyl-CoA decarboxylation (Figure 8) or other mechanisms has not been established. Product(s) of the *yfdXWUVE* operon appear to have no essential role in general acid stress responses [14] and may serve a more specialized function.

Acetic acid bacteria encounter oxalate in acidic plant-associated niches (figs, berries) and industrial settings (grains, legumes) [29]–[31]. In these environments, oxalate would be activated for decarboxylation by UctB (FCOCT), which appears to be constitutively expressed in *A. aceti* strain 1023 [25]. There may also be an endogenous source of oxalate: the acetic acid bacterium *Gluconobacter oxydans* was reported to form oxalate from glyoxylate [32] but glyoxylate oxidase (EC 1.2.3.5) is presently unassociated with any gene [33].

Bacteria are known to support few catabolic fates for oxalate/oxalyl-CoA that do not involve OXC or FCOCT [13]. Aerobic methylotrophs can reduce oxalyl-CoA to glyoxylate [34]. However, *E. coli* and *A. aceti* do not appear to possess homologues of oxalyl-CoA reductase (data not shown) and must therefore eliminate formate.

### Formate metabolism under anaerobic and aerobic conditions

In the absence of an exporter or catabolic pathway, flux through the oxalate-induced ATR cycle would lead to formate accumulation in the cytoplasm. Facultative and obligate anaerobes oxidize formate using one of several 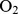-sensitive formate dehydrogenase (FDH) systems [35]. Additionally, the formate hydrogen lyase (FHL) complex, comprised of hydrogenase-3 and FDH-H, consumes protons and increases bacterial survival after anaerobic acid challenge [36]. While facultative anaerobes such as *E. coli* can eliminate formate using anaerobic respiratory enzymes [19], [37], obligate aerobes such as *A. aceti* must eliminate formate from the cytoplasm by a different method.

Known aerobic fates of formate include assimilation by formate-dependent enzymes; export, either passive or facilitated; and oxidation to 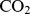. (1) Formate could be consumed by *N*-formyltetrahydrofolate synthetase (EC 6.3.4.3) [38] or the purine biosynthesis enzyme PurT (glycinamide ribonucleotide formyltransferase 2, EC 2.1.2.–) [39]. Under aerobic conditions, PurT obtains formate from an exogenous source or from PurU (*N*-formyltetrahydrofolate hydrolase, EC 3.5.1.10) [40]. Neither of these genes, however, is present in acetic acid bacteria. (2) Formate could exit the cell by passive diffusion, which is feasible in the acidic environments inhabited by acetic acid bacteria. In *E. coli*, the OxlT homologue *yhjX* may encode an inducible oxalate:formate antiporter [19], [41]. (3) Formate could be oxidized to 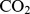 by the FDH-H homologue YdeP. *Methylobacterium extorquens* AM1 Fdh4A (48% identical to *E. coli* YdeP) supports formate oxidation during aerobic growth on methanol [42]. Moreover, *ydeP* is regulated by EvgA and has a role in acid resistance in aerobically grown enterobacteria [14], [43]. Comparable aerobic oxidation of acetate to 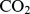 is used by *A. aceti* strain 1023 [23], [43].

### Functional inferences based on sequence and structure

CoA-transferases endow organisms with resistance pathways and the ability to use carboxylic acids as energy sources. Defining acyl-CoA substrates for these enzymes is a prerequisite for metabolic reconstructions. YfdE, YfdW, and other class III CoA-transferases have considerable sequence divergence, consistent with differing enzymatic functions. While members of the ACOCT family support both ACOCT and FCOCT activities (the latter with reduced specific activity), no member of the FCOCT family supports ACOCT activity [12], [25], [45]. The eight structurally characterized members of the class III CoA-transferase superfamily represent at least four families (Figure 5 and Figure S6), including some with no assigned functions. Differentially conserved active site loops (e.g., the ACOCT GNxH loop and the FCOCT glycine-rich loop) may impart differential substrate specificity.

### Conclusion

ACOCT uses readily available acetyl-CoA, not formyl-CoA, to make oxalyl-CoA (Figure 8). When formyl-CoA and formate are scarce (i.e., under aerobic conditions), ACOCT primes the oxalate-induced ATR cycle with substrates for OXC and FCOCT. Organisms such as *O. formigenes* that produce more promiscuous FCOCTs [12] may not require a separate priming enzyme.

## Materials and Methods

### Chemicals, enzymes, and bacterial strains

Oligodeoxynucleotides (ODNs, Table S3) were obtained from IDT (Coralville, IA). Formyl-CoA was synthesized as described previously [25]. DNA modifying enzymes were from New England Biolabs. Chemicals were from Sigma-Aldrich (St. Louis, MO) or Fisher Scientific (Houston, TX).

*E. coli* lysogens BL21(DE3) (Stratagene, La Jolla, CA) [46] and C41(DE3) (Avidis, Saint-Beauzire, France) [47] were propagated in Luria Broth (LB) medium supplemented as needed with either ampicillin (Ap) at 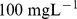 or kanamycin (Km) at 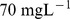.

### DNA manipulations

Genomic DNA isolated from an *E. coli* strain BW25113 derivative was used as a template for PCR with ODNs 2151 and 2152 and Vent DNA polymerase. The *yfdE* gene was cloned into the NdeI and XhoI sites of pET23a to furnish plasmid pJK560. DNA sequencing indicated this plasmid contained a silent mutation at Gly190 (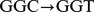) and a missense mutation at Arg232 (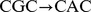), changing it to His. The missense mutation was repaired by QuikChange (Stratagene) mutagenesis using ODNs 2180 and 2181 to give YfdE expression plasmid pJK566. Other than the silent mutation at Gly190, the cloned DNA sequence matched that of *E. coli* strain K-12 *yfdE* (GenBank accession number AP009048; translation: UniProt entry P76518). The repaired *yfdE* gene was cloned into the NdeI and XhoI sites in pET28a to furnish H6YfdE expression plasmid pJK593. Quikchange mutagenesis of pJK566 using ODNs 2227 and 2228 was used to convert an ochre stop codon to Ser (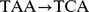), yielding YfdEH6 expression plasmid pJK594.

*A. aceti uctC* expression plasmid pJK360, encoding a 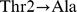 mutation, has been described previously [23]. Other than the indicated mutation, the cloned DNA sequence matched that of *A. aceti* strain 1023 *uctC* (GenBank accession number DQ668372; translation: UniProt entry A9X6P9). QuikChange mutagenesis of pJK360 using ODNs 2216 and 2217 was used to produce UctC-D177A expression plasmid pJK591.

### Analytical methods

Protein concentrations were measured using a Bradford assay kit (Bio-Rad) with crystalline bovine serum albumin as the standard [48]. Protein mass spectrometry was performed by the staff of the Washington University Mass Spectrometry Resource.

Analytical gel filtration was performed at 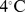 using a Pharmacia Superdex 200 Hi-load 16/60 column developed in 50 mM Tris•HCl, pH 8.0, and 100 mM KCl. Retention times for UctC (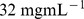 in the column buffer, adjusted to 5% glycerol) or H6YfdE (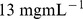 in the column buffer) were used to obtain apparent solution molecular weights as described previously [44].

### Enzyme activity assays

Substrate screening reaction mixtures contained 50 mM potassium phosphate, pH 6.7, 0.2 mM acyl-CoA, 50 mM sodium carboxylate, and 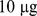 UctC or YfdE. After 1 and 10 min at 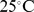, aliquots (0.1 mL) of the reaction mixture (0.5 mL) were transferred into 6.25% trichloroacetic acid (0.4 mL), vortexed briefly, and centrifuged at 16,100 g for 3 min. A zero time point was withdrawn prior to the addition of enzyme. An aliquot (0.1 mL) of each quenched reaction mixture was then subjected to HPLC analysis. Acyl-CoAs were identified by retention time.

Substrate saturation reaction mixtures contained 50 mM potassium phosphate, pH 6.7, 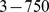 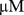 acetyl-CoA, 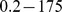 mM sodium oxalate, and 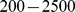 ng YfdE, H6YfdE, YfdEH6, or UctC. After 1 min at 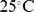, an aliquot (0.1 mL) of the reaction mixture was withdrawn and processed and analyzed as described above. A zero time point was withdrawn prior to the addition of enzyme.

CoA and acyl-CoAs were analyzed by HPLC as described previously [25]. (Acyl-)CoAs were quantitated as the sum of (acyl-)CoA and (acyl-)isoCoA (the 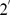-phospho isomer of CoA [49]) peak areas.

Non-enzymatic acyl-CoA hydrolysis rates were determined by fitting the time-dependent decrease in peak area, obtained by sequential injections of an authentic standard held at 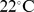, to a single-exponential decay function.

### Expression and partial purification of YfdE

LB/Ap production cultures (1 L) were inoculated using an overnight LB/Ap starter culture (20 mL) of *E. coli* BL21(DE3) cells transformed with pJK566. After growth at 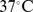 to an optical density at 600 nm (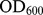) of 0.6, isopropyl 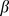-d-1-thiogalactopyranoside (IPTG) was added to a final concentration of 0.4 mM. Cells were grown an additional 4 h, harvested by centrifugation, and stored at 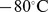. All subsequent steps were performed at 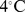. Cells were resuspended in 5 volumes of buffer S (50 mM Tris•HCl, pH 8.0, and 100 mM KCl) and disrupted by three cycles of sonication. Cell debris was removed by centrifugation at 30,000 g for 30 min. Streptomycin sulfate was added to the soluble lysate from a 10% solution to a final concentration of 1%, then allowed to stand for 10 min. After removing debris by centrifugation at 30,000 g for 60 min, the supernatant was adjusted to 35% saturation by the addition of solid ammonium sulfate (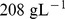) with stirring over 30 min. After stirring an additional 30 min, solids were removed by centrifugation at 30,000 g for 10 min and the supernatant was adjusted to 45% saturation by the addition of solid ammonium sulfate (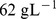) with stirring over 30 min. After stirring overnight, solids were collected by centrifugation at 30,000 g for 10 min, dissolved in a minimal volume of buffer S, and applied to a G25 column (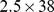 cm) equilibrated in buffer S. Fractions containing protein were pooled, concentrated by ultrafiltration to 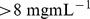 (Amicon Ultra-15, 10,000 MWCO), and applied to a DEAE-Sepharose CL-6B column (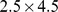 cm) equilibrated in buffer T (50 mM Tris•HCl, pH 8.0). The column was washed with one volume of buffer T and developed in a linear gradient of 0 to 0.5 M KCl (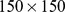 mL). Fractions containing YfdE were identified by sodium dodecyl sulfate polyacrylamide gel electrophoresis (SDS-PAGE), pooled, concentrated by ultrafiltration as described above, and applied to a Cibacron Blue 3GA column (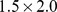 cm) equilibrated in buffer U (50 mM MES, pH 6.2, 5 mM 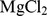, and 20 mM KCl). The column was washed with two volumes of buffer U and developed in a linear gradient of 0.02 to 1.2 M KCl (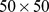 mL). Fractions (including those from the initial wash) containing YfdE were identified by SDS-PAGE, pooled, concentrated by ultrafiltration as described above, dialyzed against buffer U (4 L) overnight, and applied to a second Cibacron Blue 3GA column (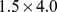 cm) equilibrated in buffer U. The column was washed with two volumes of buffer U and developed in a linear gradient of 0.02 to 1.2 M KCl (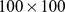 mL). Fractions containing YfdE were identified by SDS-PAGE, pooled, concentrated by ultrafiltration to 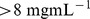, flash-frozen, and stored as single-use aliquots at 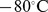.

### Expression and purification of H6YfdE and YfdEH6

LB/Km production cultures (1 L) were inoculated using an overnight LB/Km starter culture (4 mL) of *E. coli* C41(DE3) cells transformed with pJK593 or pJK594. After growth at 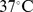 to an 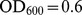, IPTG was added to a final concentration of 0.4 mM. Cells were grown for an additional 4 h, harvested by centrifugation, and stored at 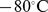. All subsequent steps were performed at 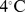. Cells (typically 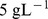 culture) were resuspended in 5 volumes of buffer S and broken by three cycles of sonication. Solids were removed by centrifugation at 30,000 g for 30 min. Streptomycin sulfate was added from a 10% solution to a final concentration of 1%, then allowed to stand for 10 min. After removing debris by centrifugation at 30,000 g for 30 min, the supernatant was adjusted to 35% saturation by the addition of solid ammonium sulfate (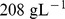) with stirring over 30 min. After stirring an additional 30 min, solids were removed by centrifugation at 30,000 g for 10 min and the supernatant was adjusted to 75% saturation by the addition of solid ammonium sulfate (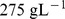) with stirring over 30 min. After stirring an additional 18 h, solids were collected by centrifugation at 30,000 g for 10 min, dissolved in a minimal volume of buffer S, and applied to a 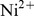-charged nitrilotriacetic acid column (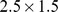 cm) equilibrated in buffer S. The column was washed with ten volumes of buffer S containing 10 mM imidazole and developed in a linear gradient of 10 to 500 mM imidazole (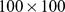 mL). Fractions containing H6YfdE or YfdEH6 were identified by SDS-PAGE, pooled, exchanged into buffer S by three cycles of diafiltration, concentrated by ultrafiltration to 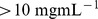 (Amicon Ultra-15, 30,000 MWCO), flash-frozen, and stored as single-use aliquots at 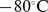.

### Expression and purification of UctC and UctC-D177A

LB/Ap production cultures (1 L) were inoculated using an overnight LB/Ap starter culture (1 mL) of *E. coli* C41(DE3) cells transformed with pJK360 or pJK591. After growth at 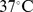 to an 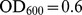, IPTG was added to a final concentration of 0.4 mM. Cells were grown at 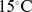 for an additional 16 h, harvested by centrifugation, and either used immediately or stored at 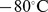. All subsequent steps were performed at 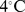. Cells (typically 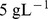 culture) were resuspended in 5 volumes of buffer B (50 mM potassium phosphate, pH 6.0, and 100 mM KCl) and broken by three cycles of sonication. Solids were removed by centrifugation at 30,000 g for 30 min. Streptomycin sulfate was added from a 10% solution to a final concentration of 1%, then allowed to stand for 15 min. After removing debris by centrifugation at 30,000 g for 30 min, the supernatant was adjusted to 45% saturation by the addition of solid ammonium sulfate (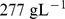) with stirring over 30 min. After stirring an additional 30 min, solids were removed by centrifugation at 30,000 g for 10 min and the supernatant was adjusted to 65% saturation by the addition of solid ammonium sulfate (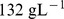) with stirring over 30 min. After stirring for an additional 30 min, solids were collected by centrifugation at 30,000 g for 10 min, dissolved in buffer C (20 mM potassium phosphate, pH 7.0), and applied to a G25 column (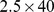 cm) equilibrated in buffer C. The column was then rinsed with several column volumes of buffer C. Fractions containing protein were pooled, concentrated by ultrafiltration to 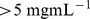 (Amicon Ultra-15, 30,000 MWCO), and applied to a DEAE-Sepharose CL-6B column (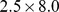 cm) equilibrated in buffer C. The column was washed with one column volume of buffer C and developed in a linear gradient of 0 to 1.2 M KCl (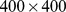 mL). Fractions containing UctC or UctC-D177A were identified by SDS-PAGE, pooled, concentrated by ultrafiltration as described above, exchanged into buffer D (50 mM Tris•HCl, pH 7.3, and 20 mM NaCl) by three cycles of diafiltration, and applied to a Reactive Red 120 column (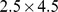 cm) equilibrated in buffer D. The column was washed with one column volume of buffer D and then developed in a linear gradient of 0.02 to 1.0 M NaCl (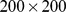 mL). Fractions containing UctC or UctC-D177A were identified by SDS-PAGE, pooled, concentrated by ultrafiltration to 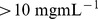 as described above, flash-frozen, and stored as single-use aliquots at 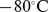.

### H6YfdE crystallization, X-ray data collection, and structure determination

Crystals were grown at room temperature (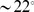
C) using the hanging drop vapor diffusion method. Drops consisted of 2 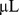 of protein solution (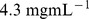 H6YfdE, 15 mM Tris•HCl, pH 8.0, and 30 mM KCl) and 2 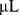 of reservoir solution. The refined reservoir solution (0.5 mL per well) contained 0.1 M Tris•HCl, pH 8.5, 0.2 M 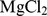, and 20% (w/v) PEG 8000. Orthorhombic crystals appeared after approximately one week and then grew to full size (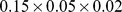 mm) over two weeks. Crystals were immersed for 2 s in reservoir solution supplemented with 15% (w/v) ethylene glycol, flash-cooled in liquid 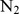 [50], and stored in liquid 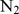. Diffraction data were collected at LS-CAT at the Advanced Photon Source (Argonne National Laboratory, Argonne, IL) and processed using the HKL2000 program suite [51]. Data collection statistics are shown in Table 3.

Molecular replacement was performed by AutoMR/Phaser [52] using a CaiB/BaiF family protein of unknown function from *Brucella suis* (PDB entry 4ed9) sharing 39% identity with YfdE. Crystallographic symmetry operators were applied to construct the dimeric search model but the protein structure was otherwise unaltered. Six subunits were placed in the asymmetric unit but were subsequently replaced with subunits of a YfdE homology model derived from the same CaiB/BaiF family protein using SWISS-MODEL [53]. Early rounds of model refinement were performed using strict noncrystallographic symmetry (NCS) restraints; rigid body, simulated annealing, and limited-memory Broyden-Fletcher-Goldfarb-Shanno (L-BFGS) coordinate optimization; and individual isotropic temperature factor refinement. Later rounds of model refinement were performed without NCS restraints and included automatic water picking. The final round of model refinement also included X-ray/stereochemistry weighting optimization. All rounds of refinement were performed using PHENIX [54]. Building was performed between rounds of refinement using Coot [55].

### Computations

SEED and MaGe genome browsers [56], [57] were used to search for *oxc* genes that have a syntenic relationship to two CoA-transferase genes as exemplified by the *A. aceti* strain 1023 *uctB–oxc–uctC–duf1275* region (GenBank accession number DQ668372).

SALIGN was used to superpose eight crystal structures and to compute structure-based sequence alignments [58]. Phylogenetic trees based on these alignments were assembled in MEGA5 [59] using the Maximum Likelihood (ML) method, with complete gap elimination. Evolutionary distances were computed using the JTT matrix-based method [60]. Bootstrap consensus trees (1000 replicates) were taken to represent the evolutionary history of the taxa analyzed [61].

COBALT [62] was used to align bacterial sequences identified by a PHI-BLAST [63] search of the RefSeq database using *E. coli* BW2952 *yfdW* (initial search pattern: G-G-N-A-G-G-G-G-Q-P-G-W) or *yfdE* (initial search pattern: P-X-[RKQ]-[ILVM]-G-N-[RKQ]-H-P) as the query sequence. A sequence logo was generated from the multiple sequence alignment using WebLogo 2.8.2 [64]. TeXshade or EPSPript were used to prepare sequence alignment figures [65], [66].

CCP4 program AreaIMol was used to compute molecular surface areas [67]. Pymol was used to prepare structural figures [68].

## Supporting Information

Figure S1 **Gel filtration profiles.** Gel filtration profile for H6YfdE (red trace) and UctC (black trace) on Superdex 200. The H6YfdE peak at 74.3 min corresponds to a solution size of 77 kDa (88 kDa expected for a dimer). The UctC peak at 72.8 min corresponds to a solution size of 86 kDa (84 kDa expected for a dimer). Absorbance values are scaled by the quantity of protein injected.
(PDF)

Figure S2
**Acyl-CoA hydrolysis half-lives.** Determination of the half-lives for spontaneous acyl-CoA hydrolysis in quenched reaction mixtures. Each solid line represents a fit of the data to the function 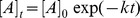. (A) Acetyl-CoA half-life is 92 h: 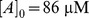 and 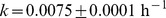. (B) Formyl-CoA half-life is 1.9 h: 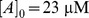 and 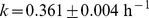. (C) Oxalyl-CoA half-life is 29 h: 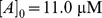 and 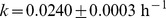. (D) Succinyl-CoA half-life is 343 h: 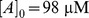 and 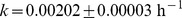.(PDF)

Figure S3 **Determination of ACOCT kinetic parameters.** (A/B) YfdE (filled circles and red traces), H6YfdE (open circles and blue traces), and YfdEH6 (filled triangles and gray traces). (C/D) UctC. Acetyl-CoA saturation curves (panels A and C) were determined at 50 mM oxalate. Oxalate saturation curves (panels B and D) were determined at 0.75 mM acetyl-CoA. Each solid line is a non-linear least-squares fit to the Michaelis-Menten equation. Kinetic parameters derived from these plots are given in Table 2.
(PDF)

Figure S4
**Stereogram of electron density in the H6YfdE active site.** The active site formed by subunits A and B is depicted from the perspective of Figure 6. Asp173 is shown in ball-and-stick rendering. Other protein atoms and waters are shown in stick and sphere rendering, respectively. The 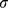 A-weighted 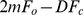 electron density map (blue mesh) is contoured at 1.2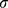 and carved with a 2.5 Å radius.(PDF)

Figure S5 **Topology diagram for H6YfdE.**
(PDF)

Figure S6
**Structure-based sequence alignment of eight class III CoA-transferase superfamily members.** This alignment corresponds to the structure superposition in Figure 2B. Secondary structure elements, except 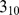 helices, are shown for H6YfdE (PDB entry 4hl6). The active site Asp is indicated by a red triangle. The locations of the ACOCT GNxH loop (PDB entry 4hl6) and FCOCT Gly-rich loop (PDB entries 1p5h, 1pt7, and 3ubm) are underlined in blue. In the YfdE sequence, large domain residues are upper-case and small domain residues are lower-case. The percent identity/similarity for each pair of sequences is given in Table S2.(PDF)

Table S1 **Mass spectrometric characterization of YfdE and UctC.**
(PDF)

Table S2 **Pairwise comparison of eight class III CoA-transferase superfamily members.**
(PDF)

Table S3 **Oligodeoxynucleotides used in this study.**
(PDF)
