# Supplementary material for: Function and X-Ray crystal structure of Escherichia coli YfdE
Source: PLoS One. 2013 Jul 23;8(7):e67901. doi: 10.1371/journal.pone.0067901 (PMC3720670; doi:10.1371/journal.pone.0067901)
Supplement: Figure S1 — Gel filtration profiles. Gel filtration profile for H6YfdE (red trace) and UctC (black trace) on Superdex 200. The H6YfdE peak at 74.3 min corresponds to a solution size of 77 kDa (88 kDa expected for a dimer). The UctC peak at 72.8 min corresponds to a solution size of 86 kDa (84 kDa expected for a dimer). Absorbance values are scaled by the quantity of protein injected. (PDF) [file pone.0067901.s001.pdf]

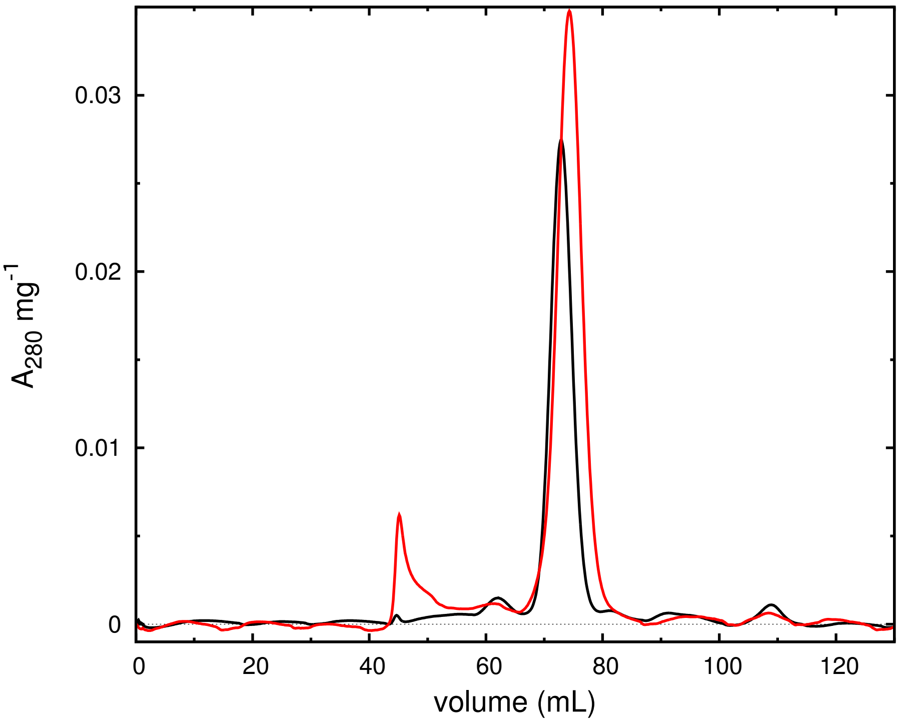

Figure S1. Gel filtration profiles. Gel filtration profile for H6YfdE (red trace) and UctC (black trace) on Superdex 200. The H6YfdE peak at 74.3 min corresponds to a solution size of 77 kDa (88 kDa expected for a dimer). The UctC peak at 72.8 min corresponds to a solution size of 86 kDa (84 kDa expected for a dimer). Absorbance values are scaled by the quantity of protein injected.
