# Supplementary material for: Function and X-Ray crystal structure of Escherichia coli YfdE
Source: PLoS One. 2013 Jul 23;8(7):e67901. doi: 10.1371/journal.pone.0067901 (PMC3720670; doi:10.1371/journal.pone.0067901)
Supplement: Figure S2 — Acyl-CoA hydrolysis half-lives. Determination of the half-lives for spontaneous acyl-CoA hydrolysis in quenched reaction mixtures. Each solid line represents a fit of the data to the function . (A) Acetyl-CoA half-life is 92 h: and . (B) Formyl-CoA half-life is 1.9 h: and . (C) Oxalyl-CoA half-life is 29 h: and . (D) Succinyl-CoA half-life is 343 h: and . (PDF) [file pone.0067901.s002.pdf]

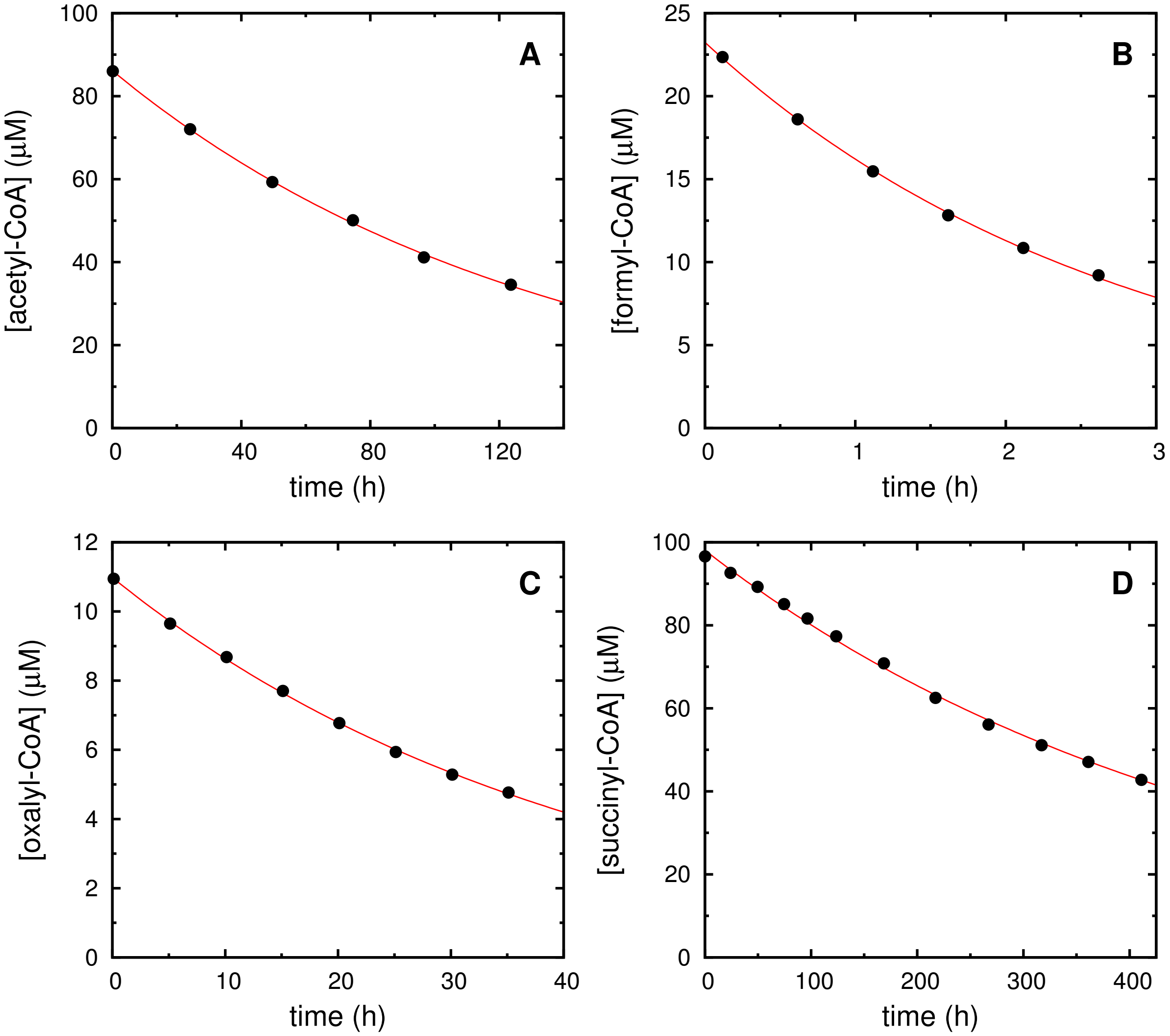

Figure S2. Acyl-CoA hydrolysis half-lives. Determination of the half-lives for spontaneous acyl-CoA hydrolysis in quenched reaction mixtures. Each solid line represents a fit of the data to the function $[A]_t = [A]_0 \exp(-kt)$. (A) Acetyl-CoA half-life is 92 h: $[A]_0 = 86$ μM and $k = 0.0075 \pm 0.0001$ h^{-1}. (B) Formyl-CoA half-life is 1.9 h: $[A]_0 = 23$ μM and $k = 0.361 \pm 0.004$ h^{-1}. (C) Oxalyl-CoA half-life is 29 h: $[A]_0 = 11.0$ μM and $k = 0.0240 \pm 0.0003$ h^{-1}. (D) Succinyl-CoA half-life is 343 h: $[A]_0 = 98$ μM and $k = 0.00202 \pm 0.00003$ h^{-1}.
