# Supplementary material for: Function and X-Ray crystal structure of Escherichia coli YfdE
Source: PLoS One. 2013 Jul 23;8(7):e67901. doi: 10.1371/journal.pone.0067901 (PMC3720670; doi:10.1371/journal.pone.0067901)
Supplement: Figure S3 — Determination of ACOCT kinetic parameters. (A/B) YfdE (filled circles and red traces), H6YfdE (open circles and blue traces), and YfdEH6 (filled triangles and gray traces). (C/D) UctC. Acetyl-CoA saturation curves (panels A and C) were determined at 50 mM oxalate. Oxalate saturation curves (panels B and D) were determined at 0.75 mM acetyl-CoA. Each solid line is a non-linear least-squares fit to the Michaelis-Menten equation. Kinetic parameters derived from these plots are given in Table 2. (PDF) [file pone.0067901.s003.pdf]

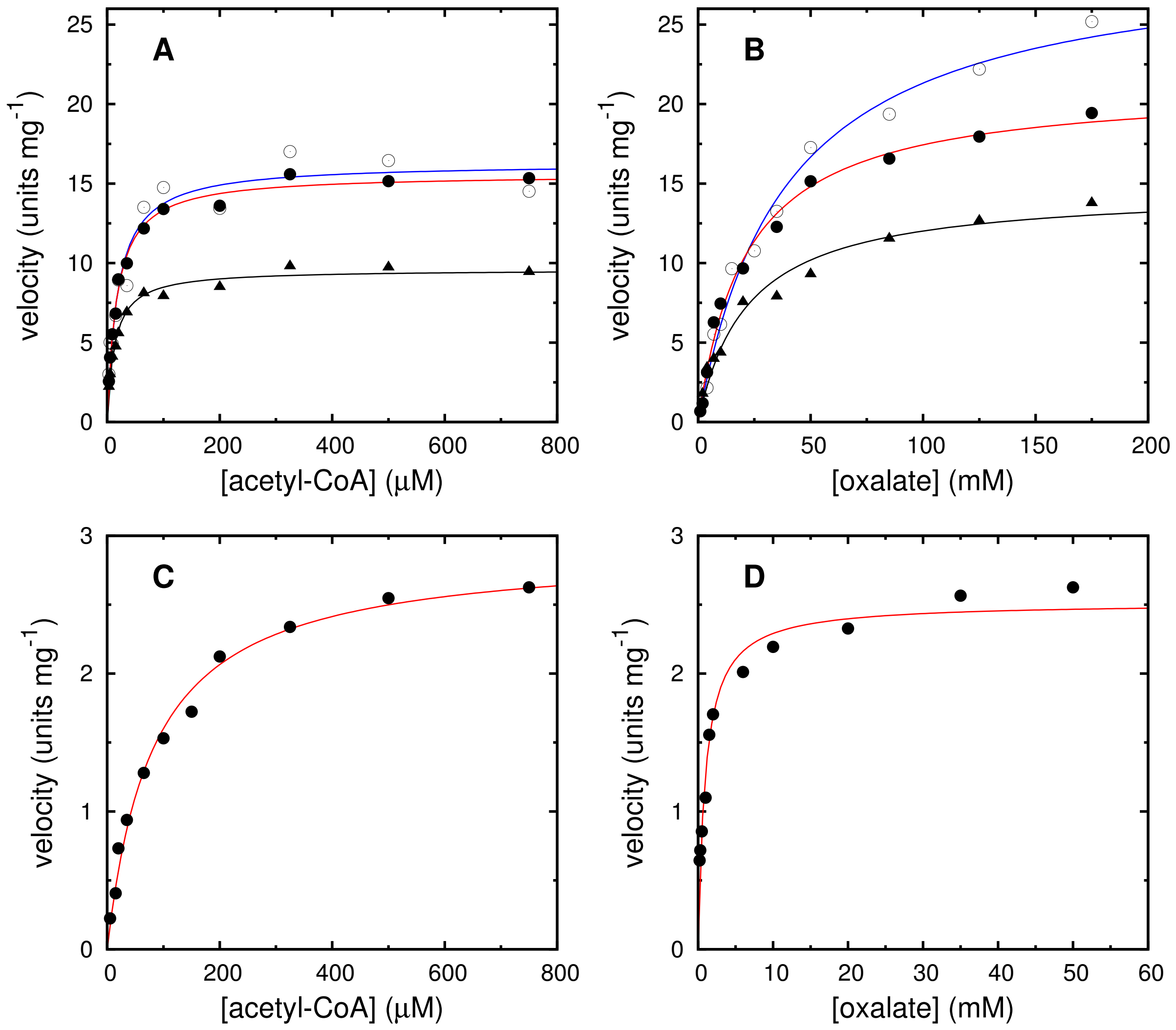

Figure S3. Determination of ACOCT kinetic parameters. (A/B) YfdE (filled circles and red traces), H6YfdE (open circles and blue traces), and YfdEH6 (filled triangles and gray traces). (C/D) UctC. Acetyl-CoA saturation curves (panels A and C) were determined at 50 mM oxalate. Oxalate saturation curves (panels B and D) were determined at 0.75 mM acetyl-CoA. Each solid line is a non-linear least-squares fit to the Michaelis-Menten equation. Kinetic parameters derived from these plots are given in Table 2.
