# Supplementary material for: Function and X-Ray crystal structure of Escherichia coli YfdE
Source: PLoS One. 2013 Jul 23;8(7):e67901. doi: 10.1371/journal.pone.0067901 (PMC3720670; doi:10.1371/journal.pone.0067901)
Supplement: Figure S4 — Stereogram of electron density in the H6YfdE active site. The active site formed by subunits A and B is depicted from the perspective of Figure 6. Asp173 is shown in ball-and-stick rendering. Other protein atoms and waters are shown in stick and sphere rendering, respectively. The A-weighted electron density map (blue mesh) is contoured at 1.2 and carved with a 2.5 Å radius. (PDF) [file pone.0067901.s004.pdf]

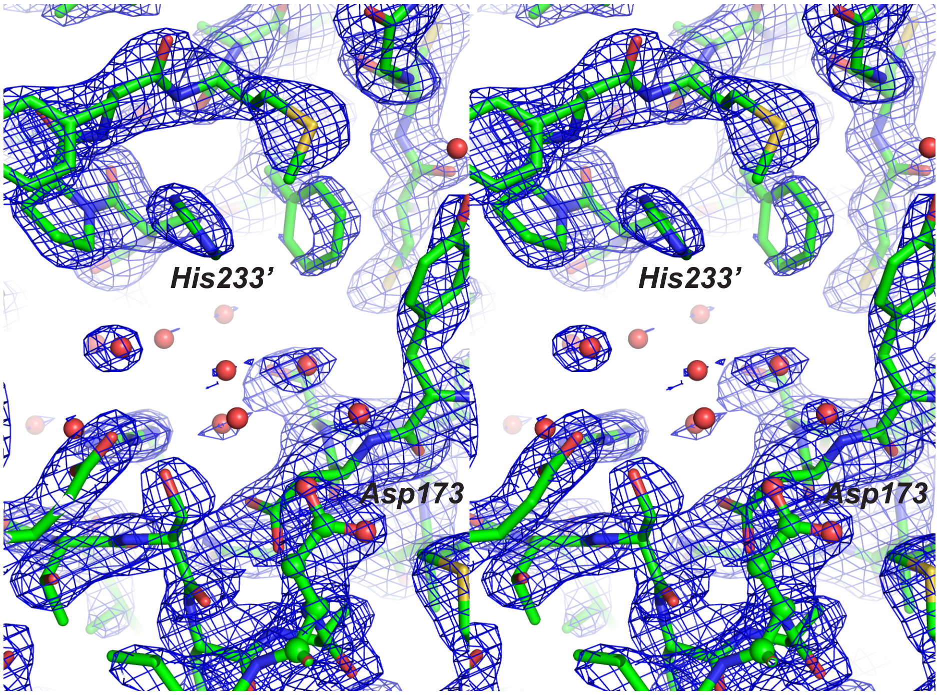

Figure S4. Stereogram of electron density in the H6YfdE active site. The active site formed by subunits A and B is depicted from the perspective of Figure 6. Asp173 is shown in ball-and-stick rendering. Other protein atoms and waters are shown in stick and sphere rendering, respectively. The σA-weighted $2mF_o - DF_c$ electron density map (blue mesh) is contoured at 1.2σ and carved with a 2.5 Å radius.
