# Supplementary figures and images for: Function and X-Ray crystal structure of Escherichia coli YfdE
Source: PLoS One. 2013 Jul 23;8(7):e67901. doi: 10.1371/journal.pone.0067901 (PMC3720670; doi:10.1371/journal.pone.0067901)

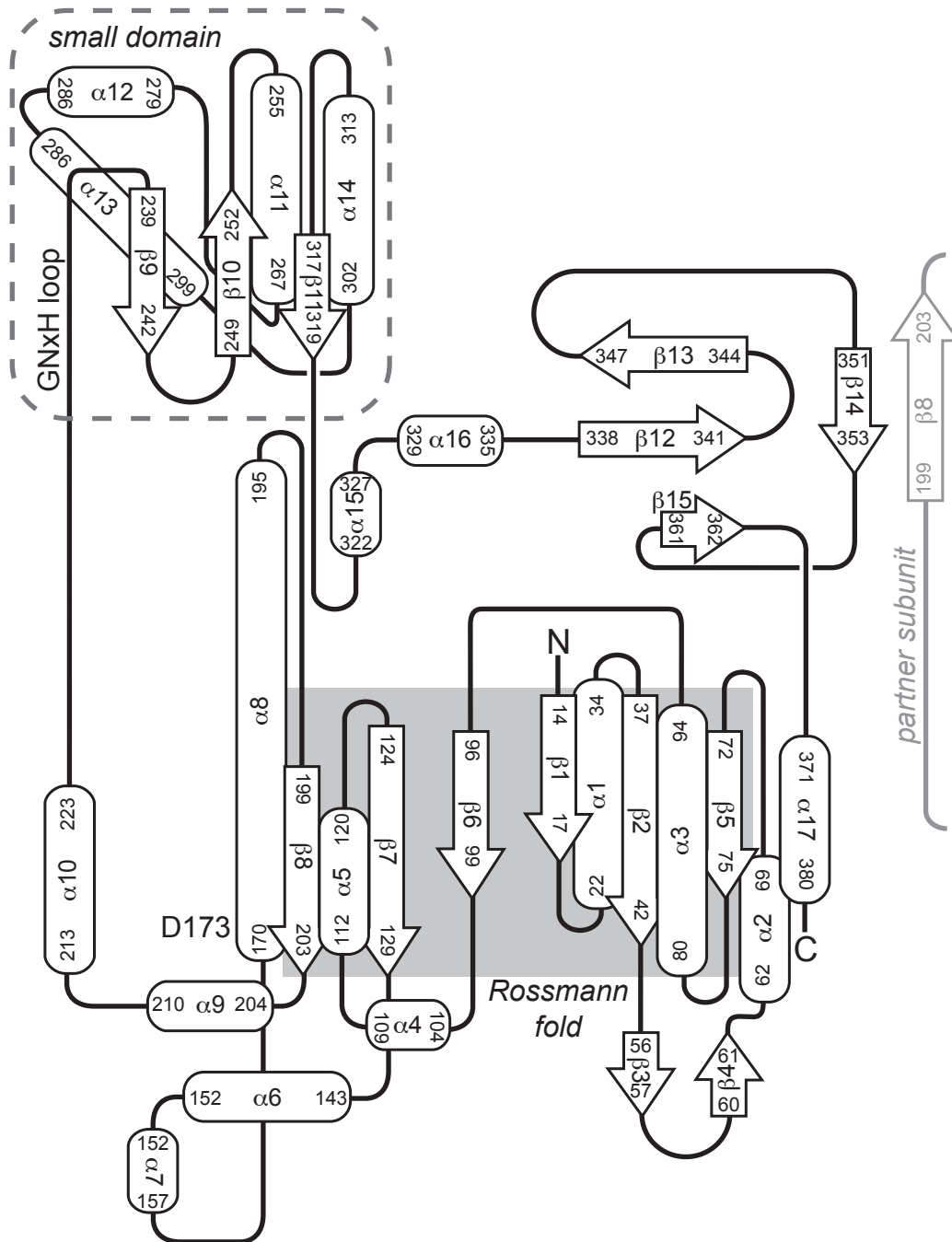

**Figure S5. Topology diagram for H6YfdE.**

Supplement: Figure S5 — Topology diagram for H6YfdE. (PDF) [file pone.0067901.s005.pdf]
