# Supplementary material for: Function and X-Ray crystal structure of Escherichia coli YfdE
Source: PLoS One. 2013 Jul 23;8(7):e67901. doi: 10.1371/journal.pone.0067901 (PMC3720670; doi:10.1371/journal.pone.0067901)
Supplement: Figure S6 — Structure-based sequence alignment of eight class III CoA-transferase superfamily members. This alignment corresponds to the structure superposition in Figure 2B. Secondary structure elements, except helices, are shown for H6YfdE (PDB entry 4hl6). The active site Asp is indicated by a red triangle. The locations of the ACOCT GNxH loop (PDB entry 4hl6) and FCOCT Gly-rich loop (PDB entries 1p5h, 1pt7, and 3ubm) are underlined in blue. In the YfdE sequence, large domain residues are upper-case and small domain residues are lower-case. The percent identity/similarity for each pair of sequences is given in Table S2. (PDF) [file pone.0067901.s006.pdf]

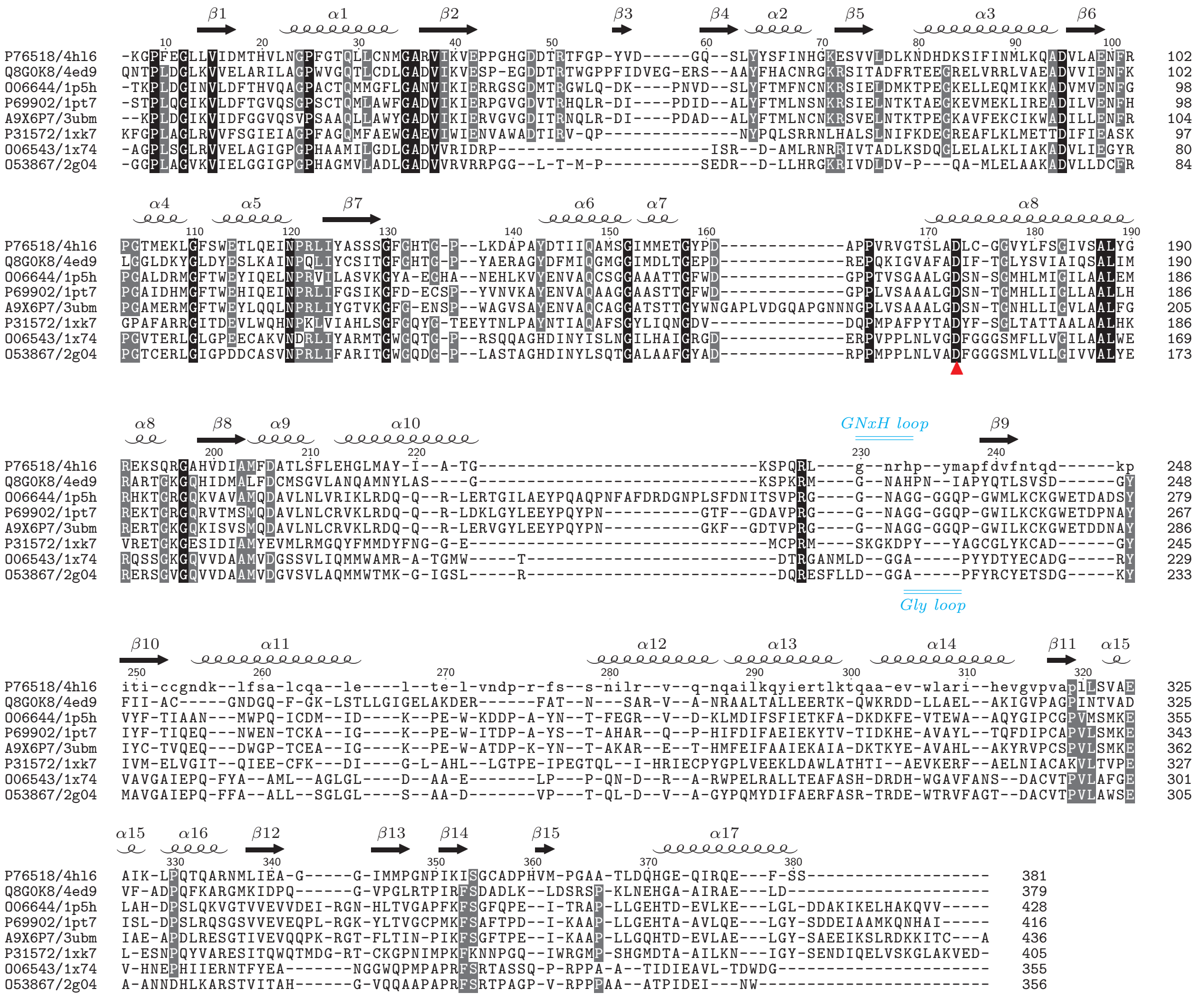

Figure S6. Structure-based sequence alignment of eight class III CoA-transferase superfamily members. This alignment corresponds to the structure superposition in Figure 2B. Secondary structure elements, except 3_{10} helices, are shown for H6YfdE (PDB entry 4hl6). The active site Asp is indicated by a red triangle. The locations of the ACOCT GNxH loop (PDB entry 4hl6) and FCOCT Gly-rich loop (PDB entries 1p5h, 1pt7, and 3ubm) are underlined in blue. In the YfdE sequence, large domain residues are upper-case and small domain residues are lower-case. The percent identity/similarity for each pair of sequences is given in Table S2.
