# Supplementary material for: Function and X-Ray crystal structure of Escherichia coli YfdE
Source: PLoS One. 2013 Jul 23;8(7):e67901. doi: 10.1371/journal.pone.0067901 (PMC3720670; doi:10.1371/journal.pone.0067901)
Supplement: Table S1 — Mass spectrometric characterization of YfdE and UctC. (PDF) [file pone.0067901.s007.pdf]

Table S1. Mass spectrometric characterization of YfdE and UctC.

Sample	Observed (Da)	Expected[a] (Da)
H6YfdE	43,702	43,703
YfdEH6	42,822	42,823[b]
	43,569	43,571[b,c]
UctC	41,708	41,709[d]
UctC-D177A	41,664	41,665[d]

[a] Computed assuming that Met1 is removed, except as noted.
[b] Retains Met1.
[c] Aspartyl-CoA thioester adduct.
[d] Thr2→Ala mutant.
