# Supplementary material for: Function and X-Ray crystal structure of Escherichia coli YfdE
Source: PLoS One. 2013 Jul 23;8(7):e67901. doi: 10.1371/journal.pone.0067901 (PMC3720670; doi:10.1371/journal.pone.0067901)
Supplement: Table S2 — Pairwise comparison of eight class III CoA-transferase superfamily members. (PDF) [file pone.0067901.s008.pdf]

Table S2. Pairwise comparison of eight class III CoA-transferase superfamily members.[a]

	ACOCT	Unknown	CaiB	Racemase		FCOCT		
UniProt	P76518	Q8G0K8	O06543	O53867	P31572	O06644	A9X6P7	P69902
PDB entry	4hl6	4ed9	1x74	2g04	1xk7	1p5h	3ubm	1pt7
4hl6	–	56.8	43.4	44.1	39.5	49.3	49	49.5
4ed9	**38.3**	–	45.3	42	42.7	47.3	49	50.7
1x74	**25.6**	**30.4**	–	74.4	42	41.1	43.8	44.1
2g04	**27.3**	**29.2**	**58.1**	–	39.8	41.7	43.8	42.9
1xk7	**22.5**	**25.1**	**24.2**	**22.2**	–	40.1	42.3	42.5
1p5h	**30.7**	**29.1**	**25.1**	**23.3**	**23.0**	–	73.8	75.4
3ubm	**31.2**	**29.0**	**26.2**	**25.6**	**25.8**	**58.5**	–	84
1pt7	**31.0**	**30.3**	**25.1**	**24.5**	**24.3**	**60.2**	**70.7**	–

[a] Pairwise percentage identities (bold font, below the diagonal) and similarities (normal font, above the diagonal) in the structure-based sequence alignment (Figure S6).
