# Supplementary material for: Function and X-Ray crystal structure of Escherichia coli YfdE
Source: PLoS One. 2013 Jul 23;8(7):e67901. doi: 10.1371/journal.pone.0067901 (PMC3720670; doi:10.1371/journal.pone.0067901)
Supplement: Table S3 — Oligodeoxynucleotides used in this study. (PDF) [file pone.0067901.s009.pdf]

Table S3. Oligodeoxynucleotides used in this study.

ODN	Sequence ($5' \rightarrow 3'$)[a]
2151	*CAT AAG CAT* ATG ACA AAT AAT GAA AGC AAA GGG
2152	*AGC ACA TCC TCG AG*T TAT GAT GAG AAC TCC TGG CGA A
2180	CCA TGT AGG GAT GGC GAT TTC CCA GAC
2181	GTC TGG GAA ATC GCC ATC CCT ACA TGG
2216	CAT CGC TTT CTG CTC TGT GCG GTG G
2217	CCA CCG CAC AGA GCA GAA AGC GAT G
2227	*GGT GGT GCT CGA G*TG ATG ATG AGA ACT C
2228	GAG TTC TCA TCA TCA *CTC GAG CAC CAC C*

[a] Vector sequences are in italics. Changes in the coding region relative to the genomic sequence are underlined.
